# Supplementary material for: Children exhibit a developmental advantage in the offline processing of a learned motor sequence
Source: Commun Psychol. 2024 Apr 15;2:30. doi: 10.1038/s44271-024-00082-9 (PMC11332225; doi:10.1038/s44271-024-00082-9)
Supplement: Supplementary file 3 — Reporting Summary [file 44271_2024_82_MOESM3_ESM.pdf]

nature portfolio

Corresponding author(s): Dr. Bradley King

Last updated by author(s): YYYY-MM-DD

Reporting Summary

Nature Portfolio wishes to improve the reproducibility of the work that we publish. This form provides structure for consistency and transparency in reporting. For further information on Nature Portfolio policies, see our Editorial Policies and the Editorial Policy Checklist.

Statistics

For all statistical analyses, confirm that the following items are present in the figure legend, table legend, main text, or Methods section.

n/a	Confirmed	
☐	☒	The exact sample size (n) for each experimental group/condition, given as a discrete number and unit of measurement
☐	☒	A statement on whether measurements were taken from distinct samples or whether the same sample was measured repeatedly
☐	☒	The statistical test(s) used AND whether they are one- or two-sided<br>*Only common tests should be described solely by name; describe more complex techniques in the Methods section.*
☐	☒	A description of all covariates tested
☐	☒	A description of any assumptions or corrections, such as tests of normality and adjustment for multiple comparisons
☐	☒	A full description of the statistical parameters including central tendency (e.g. means) or other basic estimates (e.g. regression coefficient) AND variation (e.g. standard deviation) or associated estimates of uncertainty (e.g. confidence intervals)
☐	☒	For null hypothesis testing, the test statistic (e.g. F, t, r) with confidence intervals, effect sizes, degrees of freedom and P value noted<br>*Give P values as exact values whenever suitable.*
☐	☒	For Bayesian analysis, information on the choice of priors and Markov chain Monte Carlo settings
☐	☒	For hierarchical and complex designs, identification of the appropriate level for tests and full reporting of outcomes
☐	☒	Estimates of effect sizes (e.g. Cohen's d, Pearson's r), indicating how they were calculated

Our web collection on statistics for biologists contains articles on many of the points above.

Software and code

Policy information about availability of computer code

Data collection	The tasks were implemented in the online acquisition platform Psytoolkit. The scripts used for data collection are available from the corresponding author on reasonable request.
Data analysis	Data processing was done in Matlab (version 2020b) and statistical analyses were done in SPSS Statistics 28 (IBM) and RStudio version 1.3.1093 (Posit, PBC, Boston, Massachusetts, USA) . Customized data processing code in MATLAB is made publicly available without restriction on Zenodo.

For manuscripts utilizing custom algorithms or software that are central to the research but not yet described in published literature, software must be made available to editors and reviewers. We strongly encourage code deposition in a community repository (e.g. GitHub). See the Nature Portfolio guidelines for submitting code & software for further information.

Data

Policy information about availability of data

All manuscripts must include a data availability statement. This statement should provide the following information, where applicable:

- Accession codes, unique identifiers, or web links for publicly available datasets
- A description of any restrictions on data availability
- For clinical datasets or third party data, please ensure that the statement adheres to our policy

The source data used for the results presented in this text as well as the raw data are publicly available without restriction on Zenodo.

Human research participants

Policy information about studies involving human research participants and Sex and Gender in Research.

Reporting on sex and gender	After providing informed consent/assent, participants completed an online screening questionnaire. In this questionnaire, they were asked what gender they identify with the most. The gender distribution was the following:<br>Experiment 1 (F/M) - Children: 17/16, adolescents: 12/21, young adults: 23/9, older adults: 24/8<br>Experiment 2 (F/M) - Children: 16/11, adolescents: 12/15, young adults: 21/6, older adults 18/9. All participants reported one of these two gender and none indicated to be non-binary. This information is contained in the manuscript.
Population characteristics	Healthy volunteers between 7-35 or 55-75 years-old of all genders were recruited. Participants were excluded if they: 1) reported history of medical, neurological, psychological or psychiatric conditions, 2) use of psychoactive or sleep-influencing medications, 3) indications of abnormal or irregular sleep, 4) mobility limitations of the fingers or hands, or 5) considered a professional typist or prior extensive training on a musical instrument requiring dexterous finger movements (e.g., piano, guitar).
Recruitment	Participants were recruited by advertisements on relevant websites and research databases, with no geographical restrictions.
Ethics oversight	All experimental procedures were approved by the University of Utah Ethics Committee (IRB_00136894).

Note that full information on the approval of the study protocol must also be provided in the manuscript.

Field-specific reporting

Please select the one below that is the best fit for your research. If you are not sure, read the appropriate sections before making your selection.

☐ Life sciences ☒ Behavioural & social sciences ☐ Ecological, evolutionary & environmental sciences

For a reference copy of the document with all sections, see nature.com/documents/nr-reporting-summary-flat.pdf

Behavioural & social sciences study design

All studies must disclose on these points even when the disclosure is negative.

Study description	Quantitative experimental (between-subjects design)
Research sample	Our research sample consisted of healthy volunteers (age range: 7-35 and 55-75 years, N = 224, 143 females). Note that as our experiments were conducted through a web-based data collection platform, recruitment was not constrained to a specific geographical region. Participants had no history of medical, neurological or psychiatric conditions and were free of medications. All participants reported normal sleep quality and quantity during the month and the night prior to the study. We did not include profession typists or individuals that received prior extensive training on a musical instrument requiring dexterous finger movements.
Sampling strategy	Participants were randomly sampled from the population of interest (see above). Experiment 1: for the assessment of age-group differences in initial motor sequence learning, and to detect an effect size of f = 0.3 (based on the comparison of initial learning between 9-year-olds and young adults in Adi-Japha et al., 2014), with an alpha of 0.05 and power of 0.80, the desired sample size was 128 subjects (32 per group). Experiment 2: For the assessment of age-group differences in macro-offline consolidation processes (i.e., Experiment 2), the detection of an effect size of f = 0.20 (slightly more conservative than group Adi-Japha et al., 2014), with an alpha of 0.05, a power of 0.80 and a correlation among repeated measurements of 0.25, this experiment required a sample size of 108 (27 participants per group). Note that the procedures of Experiment 1 (assessing initial motor sequence learning) were identical to the first session of Experiment 2 (assessing macro-offline consolidation). Accordingly, data from a subset of early participants from Experiment 2 (n = 42; 16 children, 19 adolescents, 4 young adults, 3 older adults) were included in the analyses of Experiment 1. Thus, these participants were included in analyses for both experiments.
Data collection	Participants completed a series of motor tasks on their personal computer from home, via the online acquisition platform Psytoolkit. Participants were blind to the study hypothesis during data collection.
Timing	Data collection started on 02/02/2021 and ended on 01/31/2023.
Data exclusions	In experiment 1, data from 7 participants were excluded from data analysis due to a failure to comply to experimental instructions (e.g., repeatedly pressing the same key; n = 3 children), missing data because of software issues (n = 1 young adult), or a failure to correctly perform the motor task (i.e., statistical outliers (> 3SD from group mean) on sequence accuracy; n = 1 child, 1 young adult, 1 older adult). Excluded participants were replaced and thus the final sample size for analyses consisted of 130 participants.<br>In experiment 2, 21 individuals were excluded from data analysis. Specifically, 17 participants failed to comply to experimental instructions (i.e., 4 children repeatedly pressed the same key and 5 children, 2 young adults and 6 older adults did not adhere to the specific schedule of the experimental sessions). Additional exclusions were due to inaccurate data because of software issues (n = 1 older adult), a lack of performance improvements across training (n = 1 young adult, 1 older adult), and an inability to correctly

perform the motor task (statistical outliers on sequence accuracy; n = 1 young adult). Similar to above, excluded participants were replaced and thus the final sample for analyses consisted of 108 participants. Note that data from the third session of one young adult and the post-learning random data of one adolescent were missing; thus, these individuals were excluded from the contrasts involving these specific task runs only.

These reasons for data exclusion were pre-established.

Non-participation	No participants dropped out experiment 1. Eight participants dropped out of experiment 2 and did not complete the full experimental protocol. No specific reasons regarding their withdrawal were provided.
Randomization	There was no random group assignment. Participants were assigned to an experimental group based on age. They were divided into the following four age groups: children (operationally defined as 7-12 years old), adolescents (13-17 years old), young adults (18-35 years old) and older adults (≥ 55 years old).

Reporting for specific materials, systems and methods

We require information from authors about some types of materials, experimental systems and methods used in many studies. Here, indicate whether each material, system or method listed is relevant to your study. If you are not sure if a list item applies to your research, read the appropriate section before selecting a response.

Materials & experimental systems

n/a	Involved in the study	
☒	☐	Antibodies
☒	☐	Eukaryotic cell lines
☒	☐	Palaeontology and archaeology
☒	☐	Animals and other organisms
☒	☐	Clinical data
☒	☐	Dual use research of concern

Methods

n/a	Involved in the study	
☒	☐	ChIP-seq
☒	☐	Flow cytometry
☒	☐	MRI-based neuroimaging
